# Biological and Microscopical Sections of the Academy of Natural Sciences

**Published:** 1873-07

**Authors:** 


### BIOLOGICAL AND MICROSCOPICAL SECTIONS OF THE ACADEMY OF NATURAL SCIENCES.

May 5, 1873.

*Director W. S. W. Ruschenberger, M. D., in the chair.*

Present—Prof. J. Carson, and Drs. J. G. Hunt. F. W. Lewis, Tyson, Allen, McQuillen, Norris, Buckingham and Richardson.

Dr. J. Gibbons Hunt made a very interesting and useful verbal communication in regard to the use of extract of hæmatoxylon in the preparation of stainings of both animal and vegetable tissues. In the course of his remarks he stated that after repeated experiments with the solution as recommended by Dr. J. W. Arnold, of New York, he had been led to modify the liquid by the addition of glycerine, which was found to render it much more satisfactory as a coloring material. The proportions which yielded, in his hands, the best results, were—

| | |
|---|---|
| Arnold's solution (of alum and ext. of hæmatoxylon), | f℥ss ; |
| Glycerine, | f℥j ; |
| Alcohol, | fʒj. |

This fluid, which, when poured out into a watch-glass for use, should be perfectly transparent, resembles carmine in that it stains the nucleus of a cell very deeply ; but it also faintly colors other portions of the cellular elements, of different degrees and tints. Like chloride of gold, it enables us to demonstrate the very finest net-works of non-medulated nerve-fibres running from nucleus to nucleus in the coat of an artery, and it possesses the great advantage over the auriferous salt, that after such staining of the nerve tissue we can still study the ultimate structure of the nervous filaments.

Dr. Hunt observed that in vegetable histology hæmatoxylon had certain advantages over either aniline, carmine, or carthamus, among which he might mention the fact that when, as is too frequently the case, we overstain a specimen, this excess of color from hæmatoxylon may be in part removed by macertion in a weak acetic acid solution.
a

